# Role of Additional Screws and Rod Fixation in Cage Loading During Oblique Lateral Interbody Fusion: A Finite Element Analysis

**DOI:** 10.3390/jcm14061890

**Published:** 2025-03-11

**Authors:** Yu-Hsuan Chung, Ming-Hsien Hu, Hong-Lin Su, Yen-Nien Chen, Heng-Chih Chang

**Affiliations:** 1 Department of Orthopedics, Show Chwan Memorial Hospital, Changhua 500, Taiwan; supersam9101005@gmail.com (Y.-H.C.); minghsienhu@gmail.com (M.-H.H.); 2 Doctoral Program in Translation Medicine, College of Life Sciences, National Chung Hsing University, Taichung 402, Taiwan; suhonglin@nchu.edu.tw; 3 Rong Hsin Translational Medicine Research Center, National Chung Hsing University, Taichung 402, Taiwan; 4 Department of Post-Baccalaureate Medicine, College of Medicine, National Chung Hsing University, Taichung 402, Taiwan; 5 Department of Life Sciences, National Chung Hsing University, Taichung 402, Taiwan; 6 The iEGG and Animal Biotechnology Research Center, National Chung Hsing University, Taichung 402, Taiwan; 7 Department of Physical Therapy, Asia University, Taichung 413, Taiwan

**Keywords:** oblique lumbar interbody fusion, lateral fixation, cage position, finite element analysis

## Abstract

**Background/Objectives**: Additional lateral fixation is a method with the potential to redistribute cage loading during oblique lumbar interbody fusion (OLIF). However, its biomechanical effects remain poorly understood. This study aimed to compare the mechanical responses of the lumbar spine following OLIF, both with and without additional lateral fixation, using a finite element (FE) analysis. **Methods**: An FE lumbar model with an OLIF cage at the L4–L5 levels was developed. A lateral fixation system comprising screws and a rod was incorporated to redistribute the cage loading and enhance spinal stability. Two OLIF cage positions—centered and at an oblique angle—were compared. **Results**: The additional lateral fixation reduced cage loading by 70% (409 to 123 N) and 72% (411 to 114 N) for the centered and oblique cage positions, respectively. Without lateral fixation, the peak equivalent stress on the cage during extension increased threefold (66 to 198 MPa) for the oblique position compared with that for the centered position. **Conclusions**: An additional lateral screw–rod fixation system is suggested as a complementary approach to the OLIF technique to mitigate endplate loading and pressure.

## 1. Introduction

Intervertebral disk degeneration is a common condition among older adults [1,2]. During the degeneration process, the disc height gradually decreases, resulting in a narrower intervertebral foramen. Eventually, the intervertebral disk loses its structural function, necessitating the use of an intervertebral spacer, also called a cage, as a replacement to support and restore the intervertebral space [3]. The use of cages is a key component of lumbar interbody fusion, a widely adopted surgical technique for treating spinal disorders [4]. Oblique lumbar interbody fusion (OLIF), first introduced by Silvestre in 2012, is a minimally invasive fusion technique designed to address disc degeneration by restoring spinal stability and maintaining intervertebral height [5]. The major advantages of OLIF include a reduced risk of postoperative trauma or bleeding due to its minimally invasive approach, enhanced spinal stability, and faster recovery, facilitated by minimized tissue disruption [5]. However, endplate fractures and cage subsidence are relatively frequent complications following OLIF surgery [6,7,8,9]. The incidence of endplate fractures or cage subsidence has been reported to be 18.7% [10,11].

To reduce loading on the endplate and cage while enhancing stability, additional fixation options, such as unilateral lateral fixation with plates and screws or bilateral posterior fixation with pedicle screws and rods, can be considered. Many studies have demonstrated the effect of additional bone plate fixation in OLIF [12,13,14], while studies involving the use of additional screws and rods are rare. In many regions, bone plates for OLIF have only recently become available, but their cost remains relatively high. Many surgeons still use bone screws and rods as an additional lateral fixation for OLIF. However, the magnitude of the contribution of screws and rods in terms of load distribution on the cage is still unclear.

The purpose of this study is to compare the mechanical responses of the lumbar spine with and without lateral fixation using screws and rods via a finite element (FE) analysis. In addition, the traditional transforaminal lumbar interbody fusion (TLIF) approach, involving a cage and posterior fixation, was used for comparison. FE modeling is a powerful tool for predicting the effects of biomechanical innovations, such as lumbar fusion approaches and related devices [15].

## 2. Materials and Methods

### 2.1. Solid Modeling

**Figure 1:**
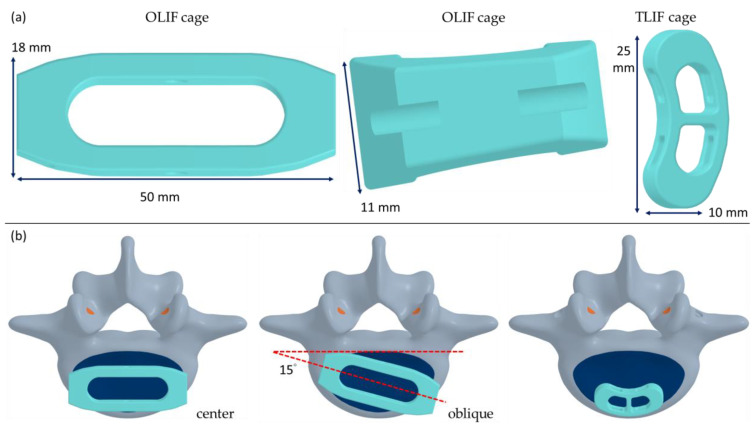
The OLIF cage and TLIF cage (**a**) used in this study. The locations of the OLIF (center and oblique) and TLIF cages (**b**).

An intact lumbar model, encompassing the L3 to sacrum segment, was developed based on computed tomographic (CT) images of a cadaveric lumbar spine. Subsequently, the intact model was refined using SolidWorks 2019 to create a symmetrical representation, incorporating both the vertebral bones and intervertebral discs, with symmetry defined along the sagittal plane. To develop the OLIF and TLIF models, the intervertebral disc between the L4 and L5 segments of the lumbar spine was removed, and an intervertebral cage was inserted into the space to provide structural support. Two distinct types of cages were utilized in this study: an OLIF cage and a TLIF cage. The dimensions of the TLIF cage were 10 mm in depth, 25 mm in width, and 10 mm in height. In contrast, the OLIF cage had dimensions of 26 mm in depth, 32 mm in width, and 10 mm in height (Figure 1a). Furthermore, two different placement locations for the OLIF cage—centered and oblique—were evaluated (Figure 1b). The centered OLIF cage was positioned at the center of the superior surface of L5, with its long axis parallel to the frontal plane. For the oblique OLIF cage, the angle between its long axis and the frontal plane was set to 15°. Additionally, supplementary lateral fixations were considered for both the OLIF and TLIF approaches at the L4–L5 segments of the lumbar spine. For the OLIF procedure, unilateral fixation with screws and a rod on the left side was employed. The screws were inserted at the center of the vertebral body on the transverse plane, positioned near the inferior surface of L4 and the superior surface of L5. The length of the screws was adjusted to ensure they extended through the opposite cortex of the vertebral body. For the TLIF procedure, bilateral posterior screws and rods were utilized. The screws were inserted into the vertebral bodies through the bilateral pedicles of the L4 and L5 segments from the posterior approach. The outer diameter of both the screws and rods was 6.5 mm. The screw length in the OLIF approach was adjusted to slightly exceed the width of the vertebral body, whereas the screw length in the PLIF approach was fixed at 55 mm.

A total of six models were employed in the simulation: the intact model, the OLIF cage standalone model (OC), the OLIF cage with lateral fixation (OC&LF), the oblique OLIF cage standalone model (O-OC), the oblique OLIF cage with lateral fixation (O-OC&LF), and the TLIF cage with posterior fixation (TC&PF).

### 2.2. Finite Element Modeling

The solid models were subsequently imported into ANSYS Workbench 2024 to predict the mechanical responses under various loading conditions. Quadratic tetrahedral elements were used to mesh the entire model, except for the rod. A total of 577,864 nodes were employed to mesh the OC model. The soft tissues of the lumbar spine were represented using tension-only springs in ANSYS Workbench. These included the anterior longitudinal ligament, posterior longitudinal ligament, ligamentum flavum, interspinous ligament, supraspinous ligament, joint capsule, and annulus fibrosus. The springs were positioned based on their anatomical locations, and their stiffness values were assigned according to data reported in the literature [16,17]. The material properties used in the simulation were also defined based on data reported in the literature [18,19] (Table 1). The number and stiffness of the spring for the annular fibrosis were defined according to a previous FE study [20,21]. The cages were made of PEEK, while the metallic implants were composed of titanium. The elastic moduli and Poisson’s ratios for PEEK and titanium were used in accordance with methods found in the literature [22,23]. All materials were modeled as isotropic and homogeneous. The contact behaviors between the bone and screw, as well as between the rod and screw, were defined as bonded. The contact behavior between the bone and cage was modeled as frictional surface-to-surface contact with a friction coefficient of 0.3 [24].

The height of the OLIF cage was slightly greater than that of the outer border of the intervertebral space, causing a slight expansion of the intervertebral space after cage insertion, particularly when the OLIF cage was obliquely placed. Subsequently, a compressive force developed on the OLIF cage prior to the application of loadings. The contact behavior between the screw and rod was defined as bonded after cage insertion. Finally, loadings were applied to the superior surface of the L3 vertebral body.

### 2.3. Convergence and Validation Tests

The number of nodes in the OC&LF increased by approximately fivefold (from 923,729 to 4,409,440) to confirm that the model had converged. Due to the complex geometry, the minimum number of nodes required to mesh the entire model was 923,729. The number of nodes increased approximately fivefold when examining the stability of the model. In the convergence test, 400 N axial compression was applied to the model, and the peak displacement at different mesh densities was compared. After convergence, for validation, the range of motion in the three major principal planes of the intact model was compared with that reported in previous cadaveric studies [25,26].

### 2.4. Boundary Condition

**Figure 2:**
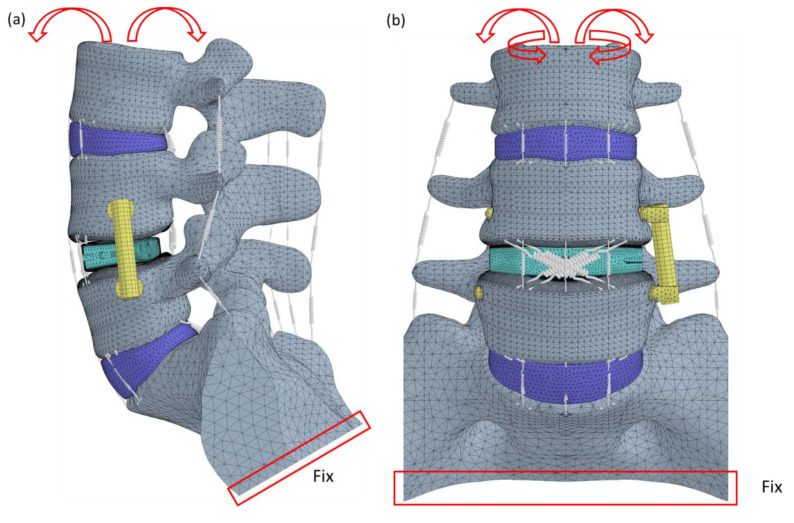
The FE model and loading conditions on the sagittal (**a**) and frontal and transverse (**b**) planes. The inferior surface of the sacrum is completely fixed (red box).

After validation, six loading conditions—flexion, extension, lateral bending to the left, lateral bending to the right, axial rotation to the left, and axial rotation to the right—were applied to the models. The loading process consisted of two steps. In the first step, a 400 N compressive force was applied to the superior surface of the L3 lumbar spine. In the second step, a 10 Nm pure moment in the six directions was also applied to the superior surface of the L3 lumbar spine [27]. The degrees of freedom at the inferior surface of the sacrum were constrained to zero, meaning no movement was allowed on that surface (Figure 2). According to Newtonian mechanics, when calculating the relative deformation among various tissues of the lumbar spine, the sacrum can be assumed to be stationary and fixed. The displacement of each vertebra, intervertebral disc, and implant can then be computed relative to the sacrum. Such a setting has been widely used in many published studies on the lumbar spine [28,29,30].

### 2.5. Index

The range of motion at each level, as well as from L3 to the sacrum, in the sagittal, frontal, and transverse planes; the loading and equivalent stress in the OLIF and TLIF cages; and the equivalent stress of the metallic screws and rods were compared. Additionally, the contact pressure on the superior surface of L5 was determined, highlighting areas of high pressure.

## 3. Results

### 3.1. Convergence and Validation

**Figure 3:**
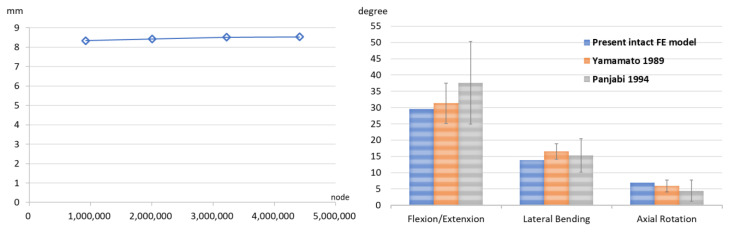
The relationship between peak displacement and the number of nodes in the convergence test (**left**), and a comparison of the range of motion in the three principal planes of the present intact model with those reported in Yamamoto’s and Panjabi’s studies [25,26] (**right**).

The peak displacement of the OC&LF model increased by only 2.4% (from 8.3 mm to 8.5 mm) when the number of nodes increased approximately fivefold. The range of motion of the current intact finite element (FE) model in the sagittal (flexion/extension), frontal (bilateral lateral bending), and transverse (bilateral axial rotation) planes was consistent with the findings reported in cadaveric studies by Yamamoto and Panjabi (Figure 3) [25,26].

### 3.2. Range of Motion

**Figure 4:**
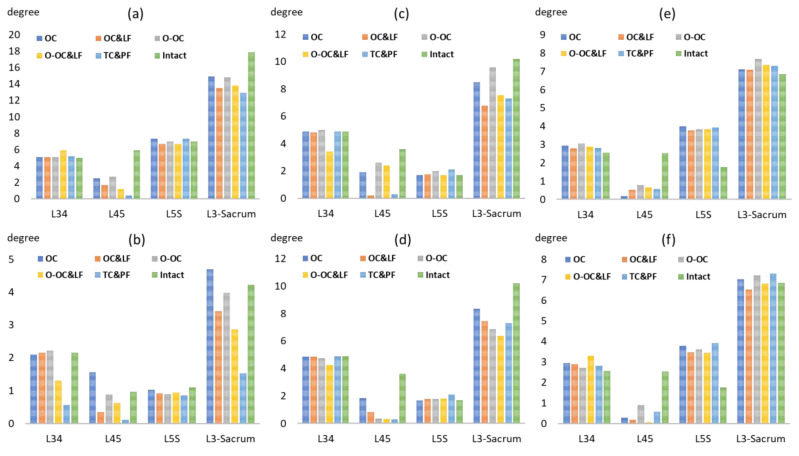
The range of motion of each level and the entire model during flexion (**a**), extension (**b**), lateral bending to the left (**c**), lateral bending to the right (**d**), axial rotation to the left (**e**), and axial rotation to the right (**f**).

The range of motion at the L4–5 levels in the models with OLIF was smaller than that in the intact model, except during extension. Compared with the intact model, the range of motion in the OC&LF model decreased by 71% (from 5.9° to 1.7°), 47% (from 3.6° to 1.9°), and 80% (from 2.5° to 0.5°) during flexion, lateral bending, and left axial rotation, respectively. At the adjacent L5–sacrum levels, the OLIF technique obviously increased the range of motion for axial rotation, while the degrees of flexion, extension, and lateral bending remained nearly unchanged (Figure 4). Notably, the range of motion in the OC model was 2.3 times greater than that of the intact model during left axial rotation.

The addition of a lateral fixation enhanced stability by reducing the range of motion, particularly in the frontal and sagittal planes, while increasing the range of motion during left axial rotation. Following the addition of the lateral fixation, the range of motion decreased by 78% (from 1.56° to 0.35°) during extension and 88% (from 1.9° to 0.22°) during left lateral bending. The oblique cage position provided a stability advantage during extension and right lateral bending but did not offer any benefits for the other types of motion. Furthermore, compared with the TLIF&PS model, the OLIF model preserved a greater range of motion for flexion, extension, and lateral bending, with no significant differences observed during axial rotation.

### 3.3. Loading and Stress on the Cage

**Figure 5:**
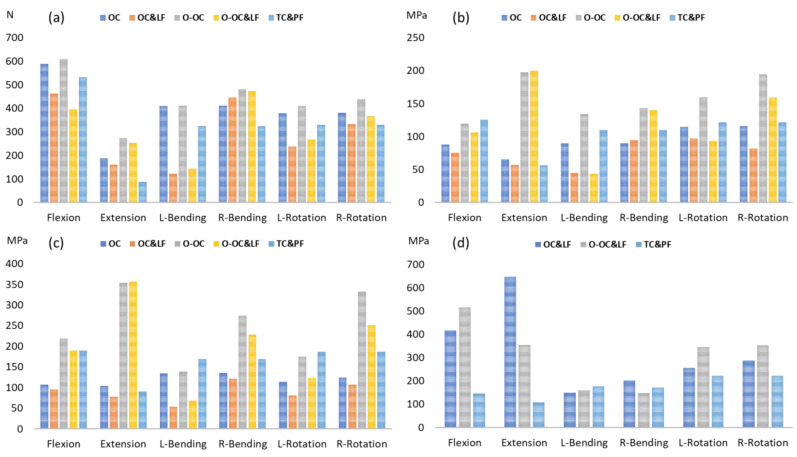
The loading (**a**) and equivalent stress (**b**) of the cage with different surgical approaches, the contact pressure on the superior surface of L5 with different surgical approaches (**c**), and the peak equivalent stress of the titanium screws and rod (**d**) with different surgical approaches.

The position of the OLIF cage obviously influenced the contact force between the bone and the cage. A contact force of 69 N was observed following cage insertion in the O-OC position. In contrast, when the OLIF cage was positioned centrally, the contact force was nearly negligible, measuring only 1 N. The additional lateral fixation redistributed the load on the cage, except in the OC&LF model during extension. During left lateral bending, the additional lateral fixation reduced the cage loading by 70% (from 409 N to 123 N) and 72% (from 411 N to 114 N) in the center and oblique cage positions, respectively (Figure 5a). Similarly, during axial rotation to the left, the additional lateral fixation decreased the cage loading by 37% (from 380 N to 238 N) and 34% (from 410 N to 269 N) in the center and oblique cage positions, respectively.

**Figure 6:**
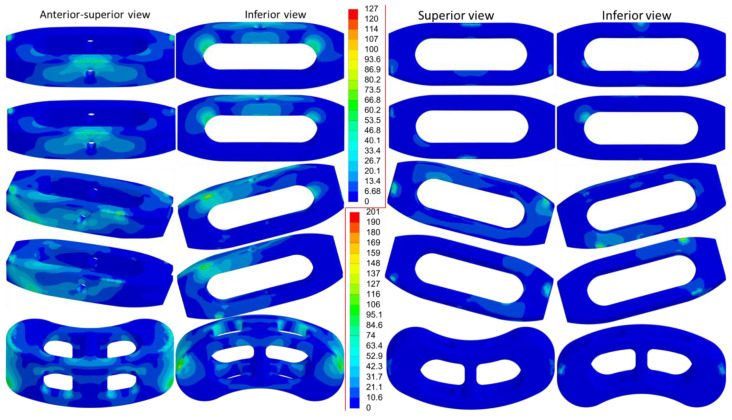
The equivalent stress in the cages during flexion (**left**) and extension (**right**). The order from top to bottom is OC, OC&LF, O-OC, O-OC&LF, and TC&PF.

**Figure 7:**
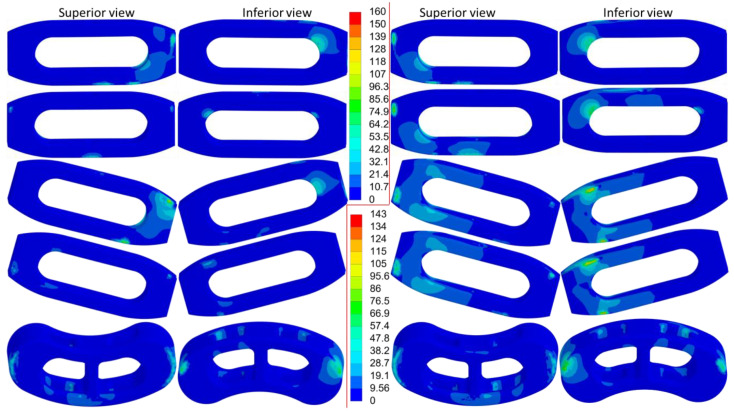
The equivalent stress in the cages during lateral bending to the left (**left**) and lateral bending to the right (**right**). The order from top to bottom is OC, OC&LF, O-OC, O-OC&LF, and TC&PF.

**Figure 8:**
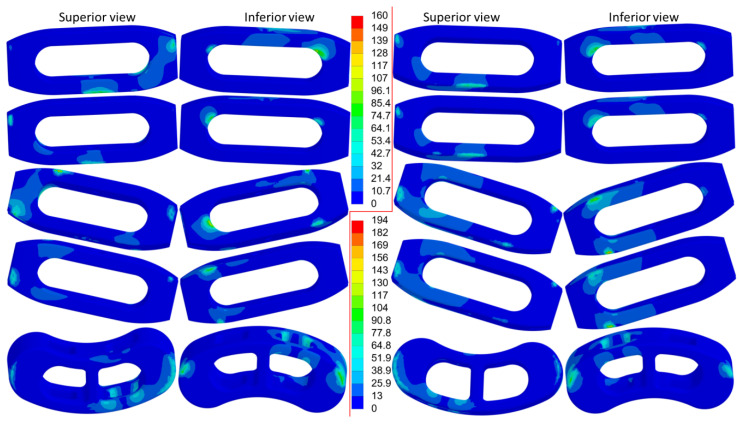
The equivalent stress in the cages during axial rotation to the left (**left**) and axial rotation to the right (**right**). The order from top to bottom is OC, OC&LF, O-OC, O-OC&LF, and TC&PF.

The distribution of equivalent stress on the cage varied across different movements, with the stress concentrated in areas in contact with the bony surfaces (Figure 6, Figure 7 and Figure 8). The peak equivalent stress on the cage decreased as the applied load was reduced. Compared with a centrally positioned OLIF cage, the oblique OLIF cage experienced higher loading and stress. Specifically, in the oblique position without lateral fixation, the peak stress on the cage during extension increased threefold, from 66 MPa to 198 MPa, compared with that in the central position (Figure 5b).

### 3.4. Contact Pressure on L5

**Figure 9:**
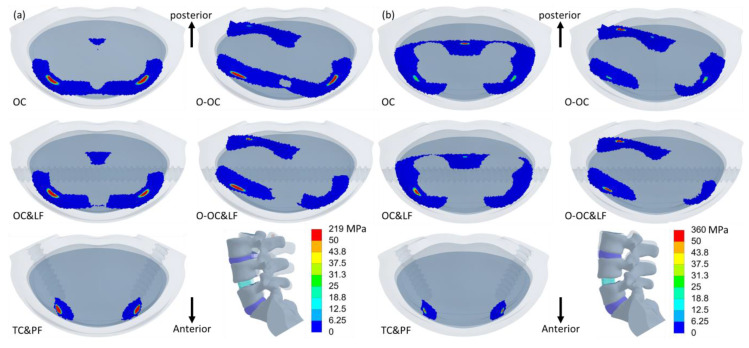
The distribution of contact pressure during flexion (**a**) and extension (**b**) on the superior surface of L5.

**Figure 10:**
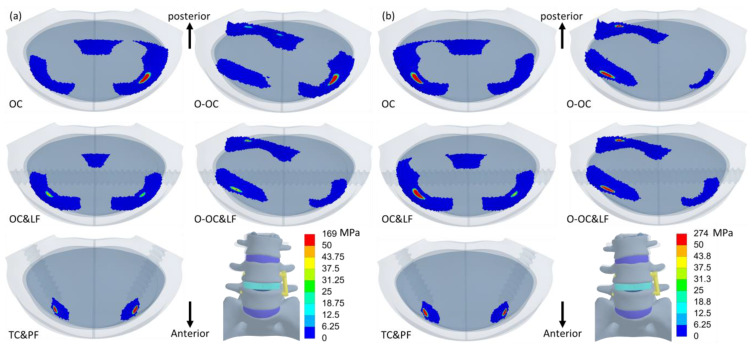
The distribution of contact pressure during bending to the left (**a**) and right (**b**) on the superior surface of L5.

**Figure 11:**
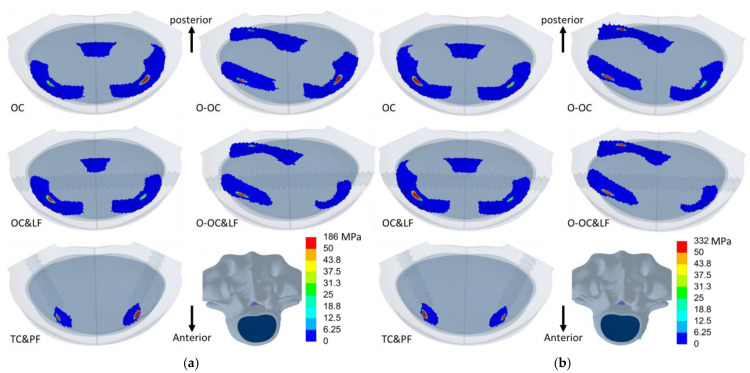
The distribution of contact pressure during axial rotation to the left (**a**) and right (**b**) on the superior surface of L5.

The cage position influences the location of the maximum contact pressure on the superior surface of L5 (Figure 9, Figure 10 and Figure 11). The addition of lateral fixation not only reduced the contact pressure on the superior surface of L5 but also altered the pressure distribution (Figure 5c). The rod partially shared the load, thereby decreasing the load borne by areas adjacent to the superior surface of L5. Consequently, the stress on the bone surface was reduced, particularly during left lateral bending. The peak contact pressure decreased by 60% (from 134 MPa to 53 MPa) with the addition of lateral fixation during left lateral bending. The OLIF cage, whether with or without lateral fixation in the central position, resulted in lower contact pressure compared with the PLIF cage with posterior fixation, except during extension. The oblique positioning of the OLIF cage resulted in lower contact pressure than the PLIF cage with posterior fixation, but only during lateral bending and left rotation.

### 3.5. Equivalent Stress of the Metallic Implants

The equivalent stress in the titanium screws and rods with the OLIF cage was obviously higher during flexion and extension than during lateral bending and rotation (Figures S1–S3). Moreover, the stress in the titanium screws and rods with the OLIF cage was substantially greater than that observed with the TLIF cage under both flexion and extension. Specifically, during extension, the stress in the titanium screws and rods in the OC&LF and O-OC&LF models was 6 and 3.3 times higher, respectively, than in the TC&PF model (Figure 5d).

## 4. Discussion

In this study, the effects of OLIF cage positioning (either central or oblique) and the use of additional lateral fixation were compared. The results demonstrated that additional lateral fixation significantly enhanced stability, while the oblique OLIF cage position was associated with an increase in peak equivalent stress. In clinical practice, surgeons should make decisions based on the patient’s condition, considering factors such as bone quality, age, and lifestyle, to achieve optimal bone fusion between the upper and lower vertebral bodies. Additionally, the potential risk of increased stress associated with different cage positions must be carefully considered.

In the sagittal plane, the lateral fixation for the OLIF cage was positioned near the center of rotation, allowing for a greater range of motion compared with the TLIF cage with posterior fixation. Additionally, the lateral fixation was located on the left side, preventing separation of the vertebral bodies on the left during right lateral bending. In contrast, the cage alone, without lateral fixation, was unable to resist tensile forces on the right side during left lateral bending. The posterior pedicle screws were bilaterally placed, providing symmetrical support and resulting in the same effect during lateral bending to both the left and right sides. In the transverse plane, all the fusion approaches evaluated in this study effectively stabilized the lumbar segment and reduced the range of motion during axial rotation.

The comparison of the current outcomes of OLIF and TLIF approaches shows that, in the TLIF procedure, extension is significantly reduced, which substantially affects spinal biomechanics. Numerous studies have indicated that TLIF is associated with a high risk of adjacent segment degeneration [31,32,33]. Regarding lateral bending, OLIF without lateral fixation preserves spinal mobility more closely to its natural state; however, the absence of lateral fixation reduces load sharing, increasing the risk of cage subsidence. In contrast, the impact of different fixation methods on rotational mobility shows no obvious differences. Overall, both OLIF and TLIF can effectively stabilize the lumbar spine. However, OLIF has less impact on the spine because the fixation is applied at the vertebral body, which serves as the rotational center during spinal movement. As a result, OLIF has a lower impact on spinal biomechanics. Additionally, OLIF offers surgical advantages, causing less soft tissue damage than TLIF. In OLIF, the impact of placing the cage in the center versus at an oblique angle is minimal. However, due to the geometric shape of the intervertebral space, where the peripheral region is narrower than the central region, an oblique placement shifts the contact area from the center to the periphery. As a result, after implantation, the intervertebral space expands, leading to increased contact pressure. Nevertheless, since the contact area is in the stronger cortical bone of the periphery, it provides better structural support. Clinicians can determine the optimal OLIF cage placement based on the patient’s bone quality.

Overall, the use of lateral fixation with an OLIF cage reduces the magnitude of loadings on the cage. However, this adjustment increases the loading on the OLIF cage during right lateral bending, as the loading distribution is altered, causing stress to concentrate on weaker areas of the structure. During right lateral bending, the OLIF cage becomes a weak point, resulting in increased loading being transferred to the cage. Using a single rod for fixation can lead to instability, as its cylindrical structure closely resembles a one-dimensional form and its circular cross-section provides uniform resistance in all directions, limiting its effectiveness in counteracting forces. Increasing the cross-sectional area and adopting a rectangular shape can enhance resistance to forces in various directions while also providing additional sites for screw fixation. The critical factor for resisting bending is the moment of inertia, which depends on the shape of the cross-sectional area (Figure S4). Currently, numerous bone plate products are available, and their clinical outcomes are well documented. However, if bone plates are unavailable and a rod must be used, the patient needs to be clearly informed of the potential risks associated with this approach.

The superior and inferior surfaces of the OLIF cage are smooth, whereas the bony surfaces of the vertebral body are much more irregular. As a result, only a small portion of the bone and cage surfaces are in contact with each other. These limited contact areas must bear the entire load of the lumbar spine, leading to pressure concentrating in these regions. During motion, the pressure increases on the compressive side and decreases on the tensile side. In addition, the lateral fixation absorbs most of the load of the cage near the rod. This reduced loading lowers the peak pressure on the bony surface, consequently decreasing the risk of endplate fracture and cage subsidence. Compared with a centrally positioned OLIF cage, an obliquely placed OLIF cage generates more pressure. This increased pressure underscores the elevated risk of endplate fracture and cage subsidence, particularly in patients with severe osteoporosis, compromised subchondral endplates, or severe degenerative changes. To mitigate these risks, several clinical strategies can be employed, including surgical technique refinements, advancements in implant design, and patient-specific surgical planning.

Surgeons can adopt meticulous endplate preparation techniques to maximize bony contact and reduce localized stress concentrations. Preserving the subchondral endplate as much as possible during discectomy and ensuring uniform cage contact with the vertebral endplate can help distribute the axial load more evenly. Additionally, fluoroscopic or intraoperative navigation-assisted cage positioning can improve accuracy in cage placement, reducing asymmetrical loading, which might contribute to subsidence. If an oblique cage placement is unavoidable due to anatomical constraints, supplementation with lateral fixation, such as anterolateral screw and rod instrumentation, or even posterior fixation with pedicle screws, is highly recommended, in order to ensure that the construct is stable. Advancements in cage structural design can also play a crucial role in mitigating endplate stress. Using larger-footprint cages that span the entire width of the vertebral body increases the load-bearing surface and decreases pressure on any single point of the endplate. In patients with osteoporosis or poor bone quality, the use of preoperative bisphosphonates or anabolic agents such as teriparatide may enhance bone density and reduce the risk of mechanical failure [34]. Preoperative imaging, including computed tomography (CT) and magnetic resonance imaging (MRI), can help assess endplate integrity and bone density, allowing surgeons to tailor their approach based on individual patient characteristics.

The ultimate goal of lumbar fusion surgery is to achieve bone fusion, where the two adjacent vertebral bodies, previously separated, fuse to become a single unit. The PEEK cage has an elastic modulus that is closer to that of bone compared to titanium cages. However, the main drawback of the PEEK cage is its lack of bone osteointegration. Recently, titanium cages have become softer as they transition from a solid to a porous structure. The key reason for this transformation is advancements in metal 3D printing technology. Many biomechanical studies have investigated the effects of the porosity of porous titanium lumbar cages on the mechanics and stability of the lumbar spine [35,36]. In addition, animal and clinical studies have demonstrated the osteointegration effect, including in vivo bony tissue ingrowth into the pores of porous titanium lumbar cages.

The clinical study conducted by Xie et al. demonstrated the effectiveness of OLIF combined with anterolateral screw fixation in the treatment of lumbar degenerative disc disease [37]. This approach significantly improved disc and foraminal heights, reduced upper vertebral slippage, and had a minimal complication rate, with only 7.7% of cases experiencing cage subsidence. These findings are consistent with those from our finite element analysis, which highlights the importance of lateral fixation in preventing mechanical failures such as subsidence and endplate fractures. Similarly, Liu et al. found that OLIF combined with additional lateral screw and rod fixation provided robust stabilization, achieving a 95% fusion rate with minimal complications [38]. Their findings support the use of lateral fixation as a time-efficient and less invasive alternative to posterior fixation, particularly in patients with one or two affected levels. Wang et al. further validated the efficacy of OLIF with additional lateral fixation in correcting coronal and sagittal deformities [39]. Their study showed significant improvements in radiographic parameters, including the Cobb angle, lumbar lordosis, and pelvic tilt. These outcomes complement our biomechanical findings by demonstrating that lateral fixation contributes to both mechanical stability and clinical efficacy.

The integration of lateral fixation with OLIF enhances stability. Our analysis demonstrated that lateral fixation effectively reduces motion and distributes stress more evenly across the spinal construct. This finding is consistent with the clinical data from Xie et al., which highlighted reduced risks of subsidence and improved fusion rates. Both Liu et al. and Wang et al. emphasized the minimally invasive nature of OLIF combined with lateral fixation, noting its ability to reduce operative times, blood loss, and soft tissue disruption compared with traditional posterior fixation methods [37,39]. Regarding cage positioning, our finite element analysis revealed that positioning the cage in the center optimizes biomechanical stability by reducing the concentration of stress on the endplates. This finding is supported by clinical studies that highlight the importance of precise cage placement in minimizing complications such as cage subsidence [37,39]. While the combined approach of OLIF and lateral fixation shows great promise, further research is needed to validate its long-term outcomes and optimize its application. By integrating finite element analysis with clinical outcomes, this study underscores the potential of using OLIF with lateral fixation as a powerful, minimally invasive alternative method for lumbar fusion. The ability of this technique to combine mechanical stability with clinical efficacy makes it an attractive option for treating degenerative lumbar diseases and deformities.

From the perspective of clinical applicability, these findings provide valuable guidance for surgical decision making, particularly in optimizing fixation strategies to prevent complications such as subsidence and endplate fractures. The integration of lateral fixation into OLIF procedures enables the construct to be more stable without the need for additional posterior instrumentation, which may reduce overall surgical times, intraoperative blood loss, and postoperative recovery periods. This is particularly beneficial for elderly patients or those with significant comorbidities, where minimizing surgical morbidity is a key consideration. Additionally, the biomechanical validation of lateral fixation enhances confidence in using this approach across a broader patient population, including those with moderate instability or sagittal misalignment. As the technique continues to evolve, the real-world application of these findings may encourage surgeons to consider patient-specific surgical planning based on preoperative imaging and bone quality assessments.

The present simulation has some limitations due to the simplifications we implemented. First, only bony structures and ligament tissues were considered, with the ligaments simplified as one-dimensional springs. Second, the material properties of the bone and implants were assumed to be linear elastic, isotropic, and homogeneous. Although the intervertebral disc was modeled with hyperelasticity, its viscoelastic properties were not considered. Therefore, the absolute values cannot be accurately represented. However, the differences and trends among the approaches still have certain reference value for orthopedic surgeons.

## 5. Conclusions

OLIF is a popular solution for managing lumbar disc degeneration because it preserves the back muscles. During the procedure, an OLIF cage combined with additional lateral fixation is recommended to reduce endplate pressure, with screws and rods being one of the options for lateral fixation. Furthermore, positioning the OLIF cage in the center is also recommended to minimize pressure. However, the long-term effects of cage positioning and the use of screws and rods for lateral fixation need to be further studied.

## Figures and Tables

**Table 1:** The material properties for the ligaments, bones, discs, and implants used in this study.

Ligament/Material	Stiffness (N/mm)	Spring Numbers at Each Tissue	Elastic Modulus (MPa)	Poisson’s Ratio	Reference
Ligament					
Anterior longitudinal	25	3	-	-	[17]
Posterior longitudinal	20	1	-	-	[16]
Ligamentum flavum	10	2	-	-	[16]
Interspinous	5	2	-	-	[16]
Supraspinous ligaments	23	1	-	-	[16]
Intertransverse	25	1	-	-	[16]
Joint capsule	10	6	-	-	[16]
Bone					
Cortex	-	-	12,000	0.3	[18]
Cancellous	-	-	100	0.2	[18]
Endplate			1000	0.3	[18]
Disc					
Nucleus	-	-	Mooney–Rivlin 2 ParameterC10, 0.12; C01, 0.09	-	[19]
Ground substance	-	-	Mooney–Rivlin 2 ParameterC10, 0.56; C01, 0.14	-	[19]
Annulus fibrosis	8	25	-	-	[20,21]
Implant					
Titanium	-	-	110,000	0.3	[23]
PEEK	-	-	3900	0.3	[22]

## Data Availability

The data are contained within this article or its Supplementary Materials.

## Supplementary Materials

The following supporting information can be downloaded at: https://www.mdpi.com/article/10.3390/jcm14061890/s1, Figure S1: Distribution of equivalent stress on the screws and rod during flexion (a) and extension (b); Figure S2. Distribution of equivalent stress on the screws and rod during lateral bending to the left (a) and to the right (b); Figure S3. Distribution of equivalent stress on the screws and rod during axial rotation to the left (a) and to the right (b); Figure S4. Demonstration of the moment of inertia (I) for a circular area and a rectangular area.
