# Supplementary material for: Role of Additional Screws and Rod Fixation in Cage Loading During Oblique Lateral Interbody Fusion: A Finite Element Analysis
Source: J Clin Med. 2025 Mar 11;14(6):1890. doi: 10.3390/jcm14061890 (PMC11943252; doi:10.3390/jcm14061890)
Supplement: Supplementary file 1 [file jcm-14-01890-s001.zip › jcm-3427557-supplementary.pdf]

Supplemental data:

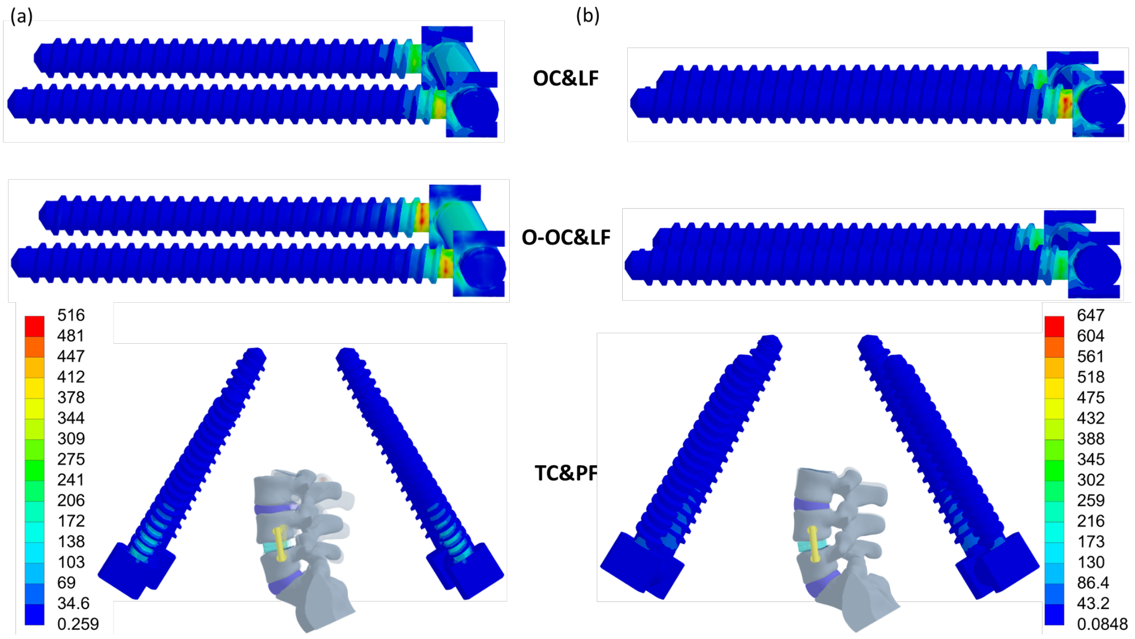

Figure S1. Distribution of equivalent stress on the screws and rod during flexion (a) and extension (b).

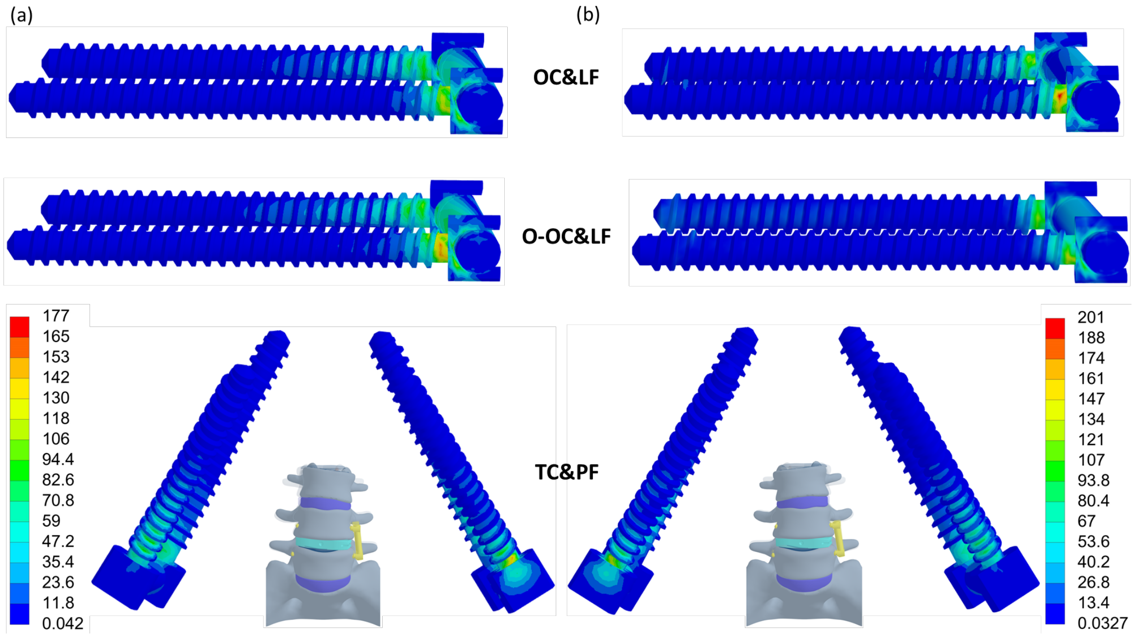

Figure S2. Distribution of equivalent stress on the screws and rod during lateral bending to the left (a) and to the right (b).

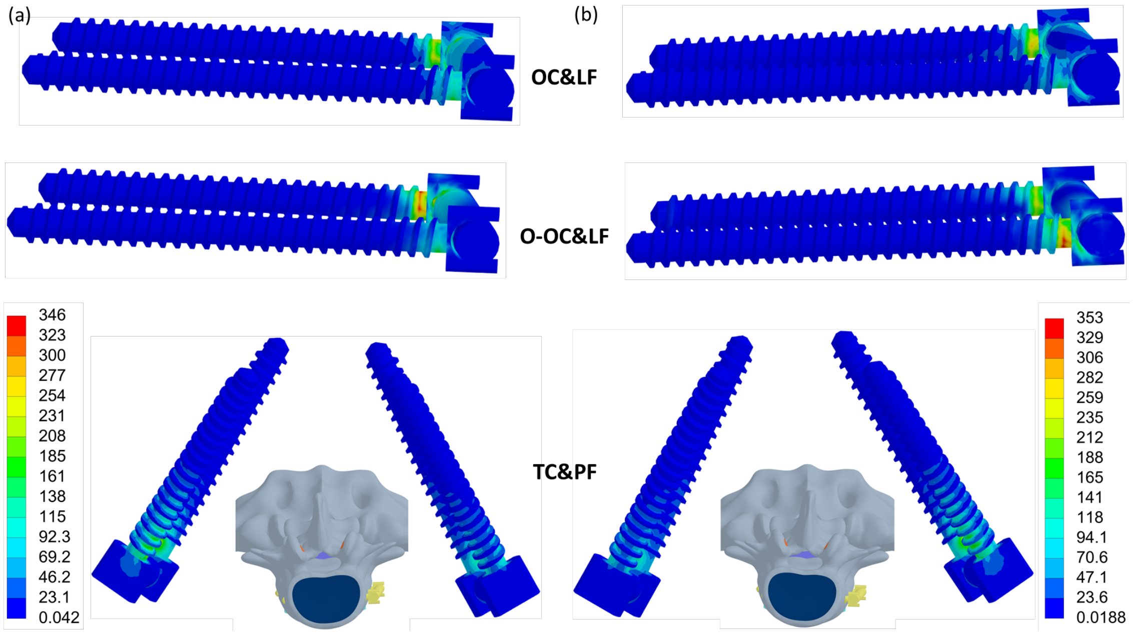

Figure S3. Distribution of equivalent stress on the screws and rod during axial rotation to the left (a) and to the right (b).

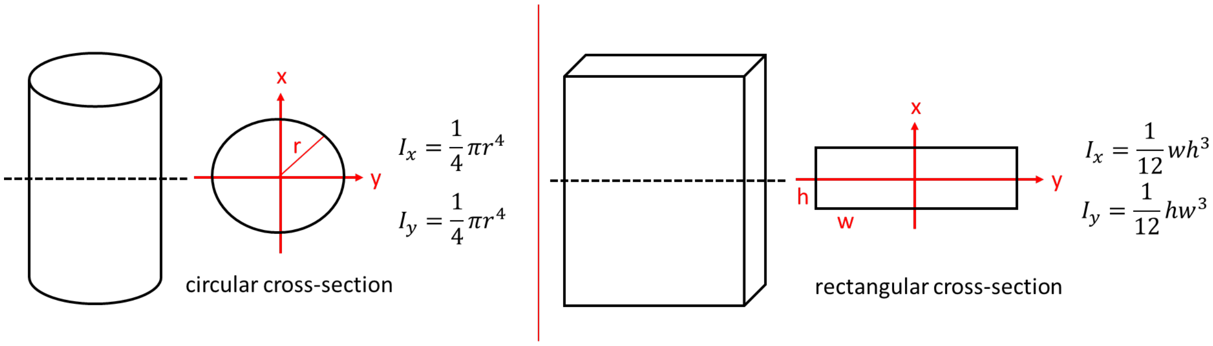

Figure S4. Demonstration of the moment of inertia (I) for a circular area and a rectangular area.
